# Supplementary material for: Association between oral microbiome and five types of respiratory infections: a two-sample Mendelian randomization study in east Asian population
Source: Front Microbiol. 2024 Apr 10;15:1392473. doi: 10.3389/fmicb.2024.1392473 (PMC11039966; doi:10.3389/fmicb.2024.1392473)
Supplement: Supplementary file 1 [file Data_Sheet_1.zip › Supplementary Materials/MR plots of saliva/Bronchitis/s__Streptococcus_mitis_AZ_mgs_2150/forest.pdf]

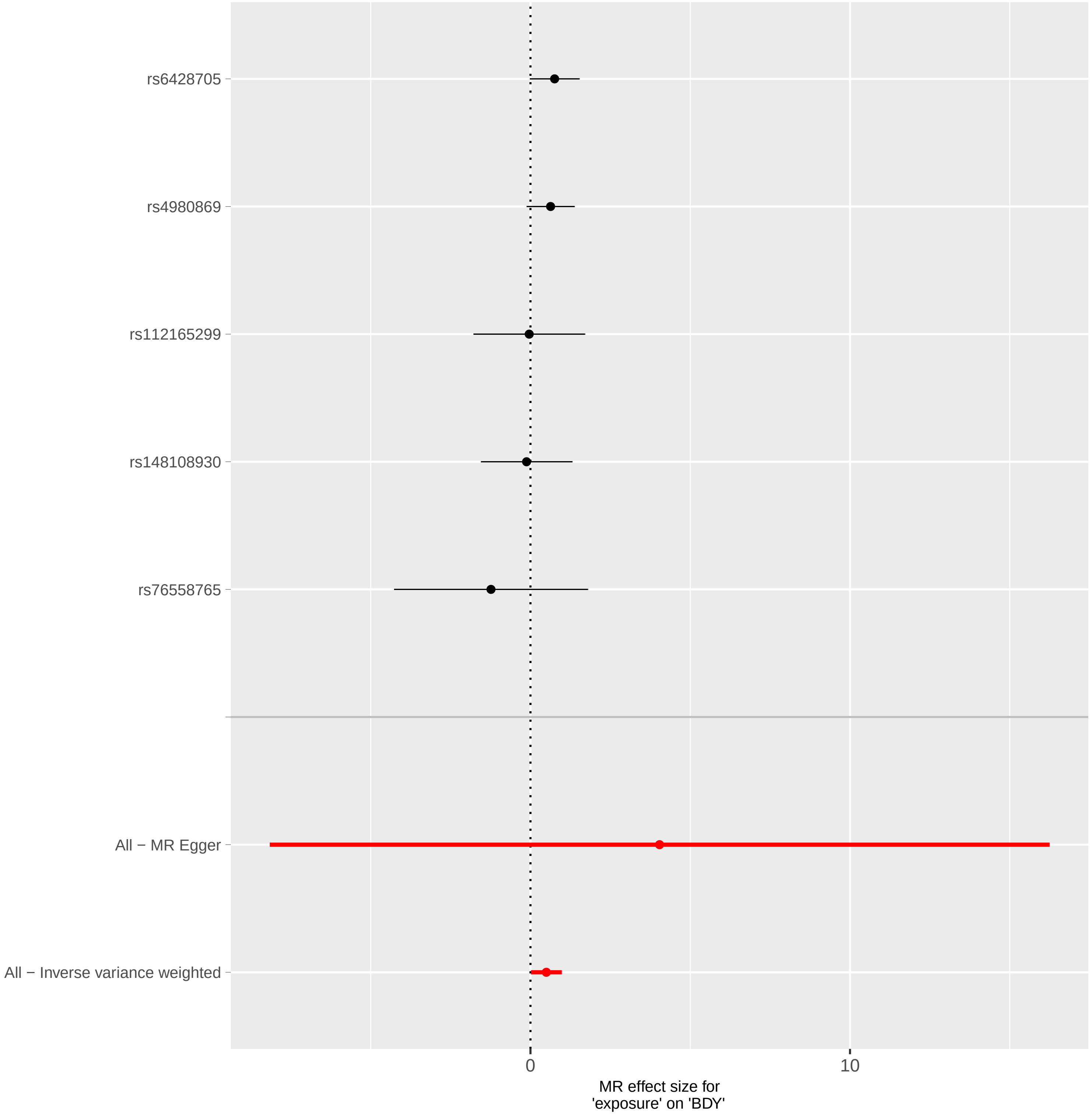
rs6428705
rs4980869
rs112165299
rs148108930
rs76558765
All − MR Egger
All − Inverse variance weighted
0
10
MR effect size for
'exposure' on 'BDY'
